# Evaluating the performance of generative adversarial network-synthesized periapical images in classifying C-shaped root canals

**DOI:** 10.1038/s41598-023-45290-1

**Published:** 2023-10-21

**Authors:** Sujin Yang, Kee-Deog Kim, Eiichiro Ariji, Natsuho Takata, Yoshitaka Kise

**Affiliations:** 1 Department of Advanced General Dentistry, College of Dentistry, Yonsei University, Seoul, Korea; 2 Department of Oral and Maxillofacial Radiology, Aichi Gakuin University, 2-11 Seuemori-Dori, Chikusa-Ku, Nagoya, 464-8651 Japan

**Keywords:** Dental pulp, Dental radiology, Endodontics, Mathematics and computing

## Abstract

This study evaluated the performance of generative adversarial network (GAN)-synthesized periapical images for classifying C-shaped root canals, which are challenging to diagnose because of their complex morphology. GANs have emerged as a promising technique for generating realistic images, offering a potential solution for data augmentation in scenarios with limited training datasets. Periapical images were synthesized using the StyleGAN2-ADA framework, and their quality was evaluated based on the average Frechet inception distance (FID) and the visual Turing test. The average FID was found to be 35.353 (± 4.386) for synthesized C-shaped canal images and 25.471 (± 2.779) for non C-shaped canal images. The visual Turing test conducted by two radiologists on 100 randomly selected images revealed that distinguishing between real and synthetic images was difficult. These results indicate that GAN-synthesized images exhibit satisfactory visual quality. The classification performance of the neural network, when augmented with GAN data, showed improvements compared with using real data alone, and could be advantageous in addressing data conditions with class imbalance. GAN-generated images have proven to be an effective data augmentation method, addressing the limitations of limited training data and computational resources in diagnosing dental anomalies.

## Introduction

The C-shaped canal configuration is a unique anatomical variation commonly found in mandibular molars. This configuration poses challenges for clinicians because of its complex canal morphology and high susceptibility to periodontal disease^1^. Proper identification and management of C-shaped canals play a crucial role in achieving successful endodontic treatment outcomes and improving the overall prognosis, because the presence of additional canals and isthmuses can lead to incomplete debridement and inadequate disinfection, compromising the long-term success of the treatment^2^. Therefore, understanding the complexities of C-shaped canals and their classification is paramount in providing optimal care for patients with these challenging anatomical variations. C-shaped canal anatomy is typically detected in clinical settings by taking a periapical radiograph or, if available, a panoramic radiograph for overall screening. However, these 2-dimensional images have limitations, such as image distortion or superimposition, which can affect diagnostic accuracy. To overcome these limitations, cone-beam computed tomography (CBCT) imaging is commonly used. CBCT produces 3D images that are reported to provide high diagnostic accuracy comparable with that obtained from conventional CT scans while having lower radiation doses^3^. However, CBCT examinations still result in significantly higher radiation doses compared with conventional panoramic or periapical radiographs, limiting their indications.

With the advancement of deep learning and computer vision, numerous studies have demonstrated the potential of this technology in dental imaging applications, including automated classification and diagnosis, as well as detection and segmentation tasks^4–6^. Studies have shown the promise of deep learning models in detecting and classifying C-shaped anatomies, achieving classification accuracies of over 90% for panoramic images^7^, CBCTs^8^, periapical images, or mixed image modalities^9^. Moreover, some studies indicate that both specialist and novice general clinicians exhibited better performance when referring to the results from deep learning models^9^. These findings suggest that the implementation of deep learning models can support clinicians in effectively classifying C-shaped canals on periapical or panoramic images and can also enhance education and training in this area. However, one common limitation shared by these studies is their relatively small dataset, typically consisting of around 1000 images. Additionally, the data exhibited high homogeneity as it was collected from only one or two institutions. Research has shown that models trained on a more diverse set of data tend to perform better for diagnosis and treatment^10^. However, traditional augmentation methods like rotation, flipping, and scaling have limitations in improving performance because they cannot alter intrinsic properties or mimic the clinical diversity of real-life datasets^11^. Consequently, novel methods that can augment the data while preserving the diversity and intrinsic properties of the real-world dataset are needed.

Generative adversarial networks (GANs) have recently emerged as a promising tool for various medical applications, including data augmentation^11^, image segmentation^12^, classification, denoising and artifact reduction^13^, super-resolution^14^, and prognosis prediction tasks^15,16^. GANs are a type of deep learning model that consist of two components: the generator and the discriminator. The generator generates new data samples, and the discriminator is responsible for distinguishing between the real and generated images. The two models are trained in an iterative and adversarial manner to enhance the generator’s ability to produce realistic data^17^. As a result, GANs can effectively address the problem of inadequate training data in medical image diagnosis and treatment models and overcome the challenge of a small dataset. However, despite the advantages that GANs offer for medical image generation, they also have drawbacks such as complexity, relatively large data requirements, and computational cost, and are thus notoriously difficult to train. Recently with continuous development and modifications in the architecture along with changes in loss functions, GANs have shown promising results in generating high-quality, diverse images with controllable styles and features compared with its most simple form (vanillaGAN)^17^. StyleGAN is a variant of progressive growing GAN (PGGAN) that introduces the style transfer function in a conditional setting. It is specially designed to generate high-quality and diverse images with controllable styles and features by adding the style transfer function in a conditional setting of the architecture of PGGANs. Nonetheless, the GANs discriminator tends to show overfit in training instances when data is scarce, hindering their ability to converge^18^. StyleGAN2-ADA was introduced in 2020 to improve upon StyleGAN2 by introducing a new data augmentation technique named adaptive discriminator augmentation (ADA). This approach improved the robustness and diversity of the generated images so they do not ‘leak’ into the generated distribution. Novel regularization approaches such as path length regularization were also chosen, leading to enhancements in the fidelity of the produced images^19,20^. The application of pretrained StyleGAN2-ADA on medical CT images achieved a high Frechet inception distance (FID) score of 5.22 (± 0.17) and 42% on the visual Turing test^21^, indicating the potential benefits of using synthetic images for data augmentation when dealing with a limited dataset and within a setting with less computational power.

Previous research on GANs in dentistry has primarily concentrated on artifact reduction or super-resolution^22–28^, modality change^29–31^, or 3D prosthesis creation^32–39^. Although some studies have employed GANs for image generation in dentistry using intraoral photographs^40^ or lateral cephalograms^41^, there have been no reported studies examining the potential of GANs for 2D radiographic imagery with limited datasets. Therefore, the aim of this study was to evaluate the quality of GAN-synthesized periapical images and evaluate the performance in diagnosing C-shaped canal anatomies. This was achieved by training StyleGAN2-ADA using periapical images of mandibular second molars with C-shaped or non C-shaped canal configurations as training data, and using pretrained weights to produce realistic periapical images. The quality of the generated images was evaluated using the FID calculation and a visual Turing test. Furthermore, the usefulness of the generated images in clinical scenarios was evaluated by performing a classification task using a convolutional neural network (CNN) to diagnose C-shaped canals.

## Methods

### Data collection

Radiographic images were selected retrospectively from a patient database at the Department of Advanced General Dentistry, Yonsei University Dental Hospital. The radiographs were of patients who underwent mandibular third molar extraction and were taken between October 2020 and October 2022. The patients were previously screened by taking periapical radiographs in the mandibular second and third molar areas. Dental CBCT examination was also performed for diagnosis or extraction risk assessment of impacted third molars, and this CBCT was used as the gold standard for classification of C- and non C-shaped canal configurations of the mandibular second molar. The CBCT images were observed by two clinical experts with over 15 and 17-years of expertise. A total of 650 patients were included after 61 were excluded for various reasons such as blurred radiographic images caused by patient movement; cropped images not showing the entire tooth; and overlapping structures such as dental implants, orthodontic appliances, plates, and screws obscuring the mandibular second molar. Cases with mandibular second molars that had undergone root canal treatment or extensive crown treatment were also excluded from the study. The patient cohort consisted of 305 males and 345 females, with an age range of 17–62 years (median = 25 years, mean age = 27.6 ± 7.3 years). Seven hundred fifty mandibular second molars were identified in total. The prevalence of C-shaped canals was 44.2%. Ultimately, a total of 1456 periapical images were prepared (non C-shaped = 803, C-shaped = 653), and cut into patches to include only the second mandibular molar as the region of interest and resized to 512 × 512 pixel sized JPG images.

This retrospective study was conducted according to the principles of the Declaration of Helsinki and was performed in accordance with current scientific guidelines. The study protocol was approved by the Institutional Review Board (IRB) of Yonsei University Dental Hospital, Seoul, Korea (approval number: 2-2023-0026). Written informed consent was waived by the IRB.

The CBCT images were acquired with the patients in a standard upright position using RAYSCAN Alpha plus (Ray Co, Hwaseong, Korea) or Pax-Zenith 3D (Vatech Co, Hwaseong, Korea) machines. The scanning parameters included a scanning time of 14 s, a field of view of 100 × 100 cm, a tube voltage of 90 kVp, a tube current of 12 mA, and a voxel size of 0.18, which were determined based on the patient’s size on the scanning device. Periapical images were taken with a tube voltage of 60 kV, a tube current of 7 mA, and an acquisition time of 0.125 s using an electric intraoral sensor.

### Acquisition of GAN images using StyleGAN2-ADA

**Figure 1:**
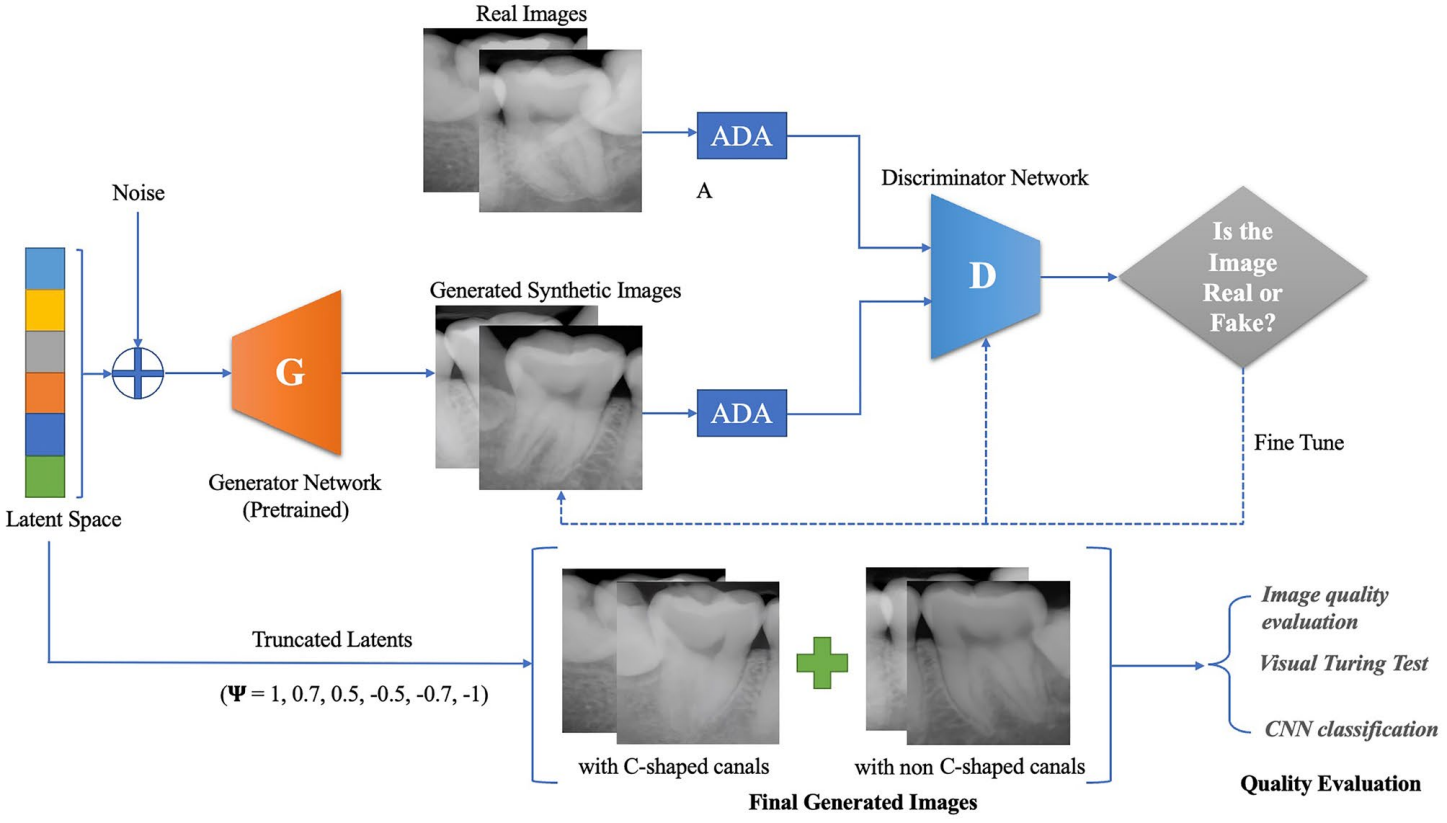
Conceptual artwork illustrating the architecture of the StyleGAN2-ADA used to generate periapical images of C-shaped and non C-shaped canal configurations, and the methods used to evaluate the quality of the generated images.

StyleGAN2-ADA was used as the generative model for this study because of its advanced capability to generate high-quality images with limited training data. The official StyleGAN2-ADA (https://github.com/NVlabs/stylegan2-ada-pytorch)^19^ and StyleGAN3 (https://nvlabs.github.io/stylegan3)^42^ repositories were used with the default parameters and without a hyperparameter search. Mirroring (horizontal flip) and ADA were enabled, and training was initialized with the official StyleGAN2 pretrained weights from Flickr-Faces High Quality Dataset (FFHQ) (https://nvlabs-fi-cdn.nvidia.com/stylegan2-ada/pretrained/). The training was performed on an NVIDIA A100-SXM graphics processing unit (GPU) with 40.0 gigabytes of GPU RAM and was executed for 600 ticks for each type of periapical image (C-shaped and non C-shaped canals), with metrics computed and weights saved every 16 ticks. The training process was repeated three times to test the algorithm stability and training convergence was evaluated by computing the average generator loss and FID improvement, and manually reviewing the generated images. The development was carried out in Pytorch version 1.19 with CUDA 11.1. All 1456 images were used for training: namely, 803 non C-shaped images and 653 C-shaped images. After training 600 images for each C shaped and non C-shaped canal, images were generated from the truncated latent space by setting the threshold Ψ, which is used to truncate and resample the latent vectors to certain numbers (Ψ = 1, 0.7, 0.5, − 0.5, − 0.7, − 1). By setting the truncation Ψ sampling can be done from a truncated normal, having values which fall outside a range to be resampled to fall inside that range. The generated images were then manually examined as thumbnails (10 × 10 pixel size) and ultimately 280 images of C-shaped mandibular second molars and 280 images of non C-shaped mandibular second molars were prepared. The conceptual architecture for training StyleGAN2-ADA and generating images is depicted in Fig. 1.

### Evaluation measures

#### Frechet inception distance (FID)

The FID serves as a metric for evaluating the quality of images produced by GANs. It gauges the similarity between two distributions, typically the distribution of real images and the distribution of generated images. A lower FID score indicates a higher level of realism in the generated images. FID is advantageous in multiple ways as it can provide a quantitative evaluation of the realism of generated images, distinguish between real and generated samples, align with human perceptual evaluations, detect distortions, and is computationally and sample efficient^43^. During the training process of StyleGAN2-ADA in generating periapical image patches, the FID was continuously monitored. The analysis focused on examining the lowest FID score that was achieved during training the GAN model. The FID was calculated five times (n = 5) using different random seeds and the average (± standard deviation) was calculated for both c and non c-shaped canal images.

#### Visual Turing test

To validate the perceptual quality of the generated images, a total of 100 images consisting of 50 generated periapical images of mandibular molars (25 with C-shaped canal configuration, 25 with non C-shaped canal configuration) which seemed real at first glance were randomly selected, and 50 real images (likewise, 25 with C-shaped canal configuration, 25 with non C-shaped canal configuration) were added. The 100 images were rearranged through random shuffling and reordering. These images were evaluated by two radiologists, each of whom had over 15 years of experience. Neither observer had any experience with synthesized periapical images. The test was performed by showing images one by one in a Google Form-based interface and the images were displayed in the same order for each observer. The observers were informed that there were 50 real and 50 generated images each, and 50 mandibular second molars with C-shaped canals and 50 with non C-shaped canals. The observers were allowed to provide only a single response to each question, without the option to revisit previous questions. They were instructed to determine whether the displayed image was genuine or synthesized. The accuracy, sensitivity, and specificity of the test results were then assessed. Fleiss kappa was used to evaluate the interobserver agreement in the visual Turing test.

#### Predictive performance based on CNN classification

EfficientNet^43^ was used for subsequent classification to evaluate the validity of the synthesized images generated by GAN. The pretrained EfficientNet-B0 which was trained on over a million images from the ImageNet database was employed. The weights from this pretraining process were used, (https://pytorch.org/hub/nvidia_deeplearningexamples_efficientnet/) and Pytorch 2.0 with CUDA 11.8 was used for development.

Six different scenarios were constructed, and the classification performances were compared. The first scenario (A) was trained only on real data (280 real images), whereas the second scenario (B) was trained only on GAN data (280 generated images). The third scenario (C) was trained on double the number of real data (560 real images) and the fourth scenario (D) was trained with a mix of real and GAN data (280 real images + 280 generated images). The fifth scenario (E) was trained on real data with class imbalance (280 real images; 56 C-shaped), and the sixth scenario (F) was trained by adding 168 synthetic images of the minority class (C-shaped) to balance the training subset with real data. The class ratio between C-shaped and non C-shaped images was equal (5:5) in scenarios A to D and F, whereas scenario E had a class imbalance of 2:8 (C vs non C) that would correspond to the real prevalence of C shaped canal configurations in the Asian population^44^. The validation and test datasets used in the study consisted entirely of real periapical images. More specifically, the validation set comprised a total of 80 real images with 40 images belonging to the C-shaped category and 40 images belonging to the non C-shaped category. The test set comprised a total of 40 real images with 20 images belonging to the C-shaped category and 20 images belonging to the non C-shaped category. Each scenario underwent five folds of trials. To achieve this, five sets of validation and testing datasets were initially prepared for each fold. Subsequently, the training data were randomly selected for each fold. In scenarios A, C, D, E, and F, a total of 280 real images were assigned to the training dataset while maintaining specific matching ratios (A, B, D: 50% C-shaped, E, F: 20% C-shaped). Without data augmentation as in scenarios C, D, and F, the ratio between training, validation, and test was set to 7:2:1. Particular attention was given to ensure that the training, validation, and test datasets did not include the same images. Moreover, the validation and test sets for each fold were also ensured to have no overlapping items. The images were then trained on EfficientNet for binary classification of C- or non C-shaped canals. The accuracy, precision, recall, specificity, false positive rate (FPR), false negative rate (FNR), and the area under the receiver operating characteristic curve (AUROC) was calculated. To assess the effectiveness of data augmentation using GAN-generated images, scenarios A and D were compared using the chi-square test (specifically, McNemar’s test). Similarly, scenarios E and F were compared to evaluate the effectiveness of GAN-generated images in addressing imbalanced dataset situations. The null hypothesis stated that there would be no significant difference between each of the two scenarios. The significance level (alpha) was set to 0.05.

### Ethics approval and consent to participate

This study was approved by the Institutional Review Board (IRB) of Yonsei University Dental Hospital (approval no. 2-2023-0026). Written informed consent was waived by the IRB.

## Results

The average FIDs (± standard deviation), n = 5, for synthesized periapical images were 72.762 (± 0.723) and 61.373 (± 3.035) for images with C shaped and non C-shaped canal configurations, respectively.

The results of the visual Turing test are shown in Table 1 with mean accuracy, sensitivity, and specificity. The accuracies were 0.490 and 0.590, sensitivities were 0.429 and 0.592, and specificities were 0.549 and 0.588 respectively for each observer. The Fleiss kappa was low (κ = 0.28) indicating poor interobserver agreement. The overall results indicate that the radiographic images generated by GAN were difficult to decipher and showed no notable difference when compared with real radiographs.

**Table 1:** Average assessment results of the two observers on the visual Turing test. The Fleiss kappa coefficient was found to be low (κ = 0.28), indicating a poor level of agreement between the observers.

	Accuracy	Sensitivity	Specificity
Observer 1	0.490	0.429	0.549
Observer2	0.590	0.592	0.588
	Interobserver agreement rate	κ = 0.28	

Table 2 presents the predictive performance of the classification models used in scenarios A to F. The classification model’s performance was weakest in scenario B where a limited number of only synthetic images from GAN were used. The accuracy, sensitivity, and specificity were 0.715 ± 0.095, 0.810 ± 0.134, and 0.620 ± 0.268 respectively. Scenarios C and D yielded better performances as the training data was augmented with either real or synthetic images, resulting in increased accuracy, sensitivity, and specificity. Scenario C had an accuracy of 0.845 ± 0.069, sensitivity of 0.810 ± 0.089, and specificity of 0.880 ± 0.084 with training data augmentation using real images. Scenario D had an accuracy of 0.890 ± 0.065, sensitivity of 0.910 ± 0.055, and specificity of 0.870 ± 0.120 with training data augmentation using synthesized images. A t-test indicated a significant difference between scenarios C and D only in sensitivity (p = 0.034) and FNR (p = 0.034) (Supplementary Table 1: T-test results). In scenarios E and F, performance was better in scenario F where the class imbalance was resolved by adding GAN-synthesized radiographs. The accuracy and specificity values were 0.855 ± 0.048 and 0.800 ± 0.050 for scenario F, and 0.800 ± 0.108 and 0.660 ± 0.219 for scenario E respectively. However, sensitivity was higher in scenario E (0.940 ± 0.065) than in scenario F (0.910 ± 0.102).

**Table 2:** The predictive performance of the classification models employed in scenarios A to F. (*FPR:* false positive rate, *FNR:* false negative rate).

Scenario	Accuracy	Sensitivity	Specificity	Precision	FPR	FNR
A Trained on real data	0.800	0.850	0.750	0.773	0.250	0.150
	0.900	0.850	0.950	0.944	0.050	0.150
	0.800	0.650	0.950	0.929	0.050	0.350
	0.875	0.750	1.000	1.000	0.000	0.250
	0.650	0.900	0.400	0.600	0.600	0.100
Average	**0.805**	**0.800**	**0.810**	**0.849**	**0.190**	**0.200**
Standard deviation	**0.097**	**0.100**	**0.248**	**0.163**	**0.248**	**0.100**
Variance	**0.009**	**0.010**	**0.062**	**0.027**	**0.062**	**0.010**
B Trained on generated data	0.850	0.750	0.950	0.938	0.050	0.250
	0.725	0.900	0.550	0.667	0.450	0.100
	0.625	0.700	0.550	0.609	0.450	0.300
	0.625	1.000	0.250	0.571	0.750	0.000
	0.750	0.700	0.800	0.778	0.200	0.300
Average	**0.715**	**0.810**	**0.620**	**0.712**	**0.380**	**0.190**
Standard deviation	**0.095**	**0.134**	**0.268**	**0.148**	**0.268**	**0.134**
Variance	**0.009**	**0.018**	**0.072**	**0.022**	**0.072**	**0.018**
C Trained on augmented real data	0.800	0.700	0.900	0.875	0.100	0.300
	0.875	0.800	0.950	0.941	0.050	0.200
	0.950	0.950	0.950	0.950	0.050	0.050
	0.775	0.800	0.750	0.762	0.250	0.200
	0.825	0.800	0.850	0.842	0.150	0.200
Average	**0.845**	**0.810**	**0.880**	**0.874**	**0.120**	**0.190**
Standard deviation	**0.069**	**0.089**	**0.084**	**0.077**	**0.084**	**0.089**
Variance	**0.005**	**0.008**	**0.007**	**0.006**	**0.007**	**0.008**
D Trained on real + generated data	0.900	0.850	0.950	0.944	0.050	0.150
	0.925	0.950	0.900	0.905	0.100	0.050
	0.975	0.950	1.000	1.000	0.000	0.050
	0.825	0.950	0.700	0.760	0.300	0.050
	0.825	0.850	0.800	0.810	0.200	0.150
Average	**0.890**	**0.910**	**0.870**	**0.884**	**0.130**	**0.090**
Standard deviation	**0.065**	**0.055**	**0.120**	**0.098**	**0.120**	**0.055**
Variance	**0.004**	**0.003**	**0.015**	**0.010**	**0.015**	**0.003**
E Trained on imbalanced real data	0.675	0.900	0.450	0.621	0.550	0.100
	0.925	1.000	0.850	0.870	0.150	0.000
	0.700	1.000	0.400	0.625	0.600	0.000
	0.850	0.950	0.750	0.792	0.250	0.050
	0.850	0.850	0.850	0.850	0.150	0.150
Average	**0.800**	**0.940**	**0.660**	**0.751**	**0.340**	**0.060**
Standard deviation	**0.108**	**0.065**	**0.219**	**0.121**	**0.219**	**0.065**
Variance	**0.012**	**0.004**	**0.048**	**0.015**	**0.048**	**0.004**
F Trained on data balanced by adding synthesized data	0.825	0.900	0.750	0.783	0.250	0.100
	0.850	0.900	0.800	0.818	0.200	0.100
	0.925	1.000	0.850	0.870	0.150	0.000
	0.875	1.000	0.750	0.800	0.250	0.000
	0.800	0.750	0.850	0.833	0.150	0.250
Average	**0.855**	**0.910**	**0.800**	**0.821**	**0.200**	**0.090**
Standard deviation	**0.048**	**0.102**	**0.050**	**0.033**	**0.050**	**0.102**
Variance	**0.002**	**0.011**	**0.003**	**0.001**	**0.003**	**0.011**

Significant values are in bold.

**Figure 2:**
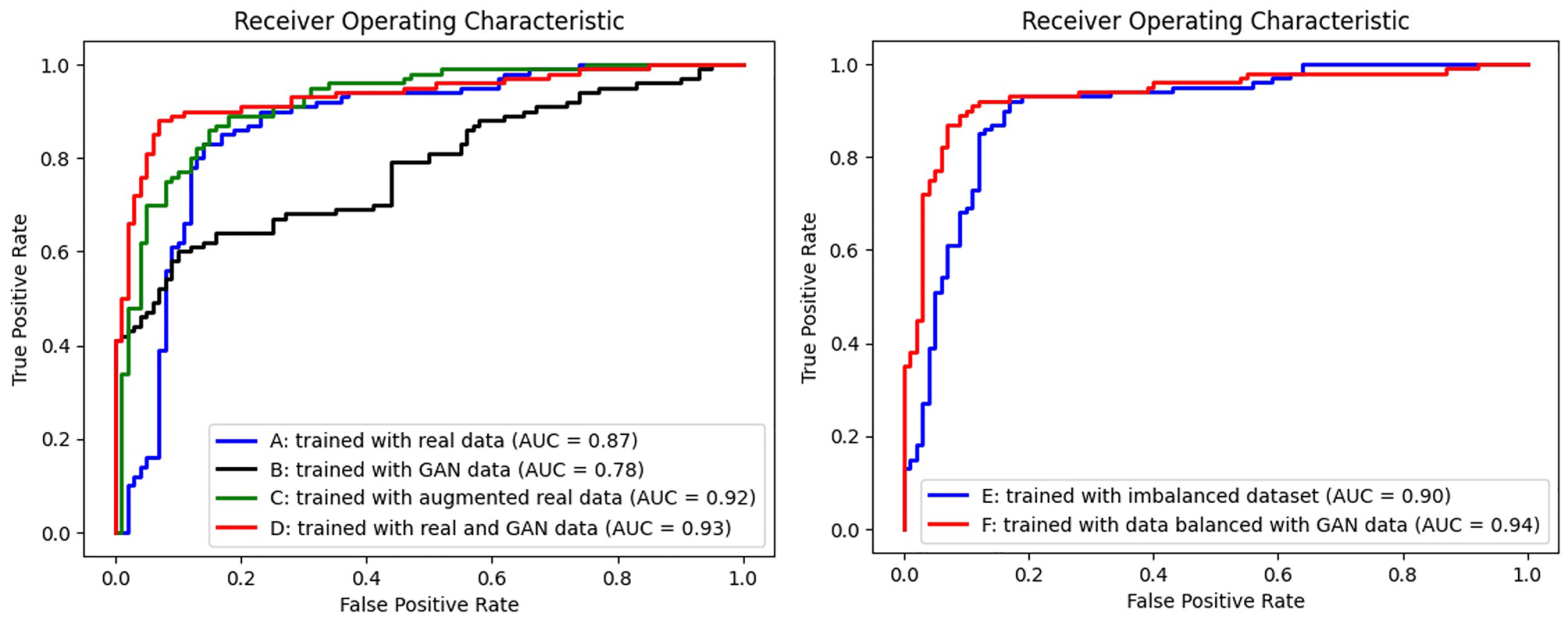
Receiver operating characteristic (ROC) curves and the corresponding area under the ROC curve (AUROC) values for scenarios A to F.

**Figure 3:**
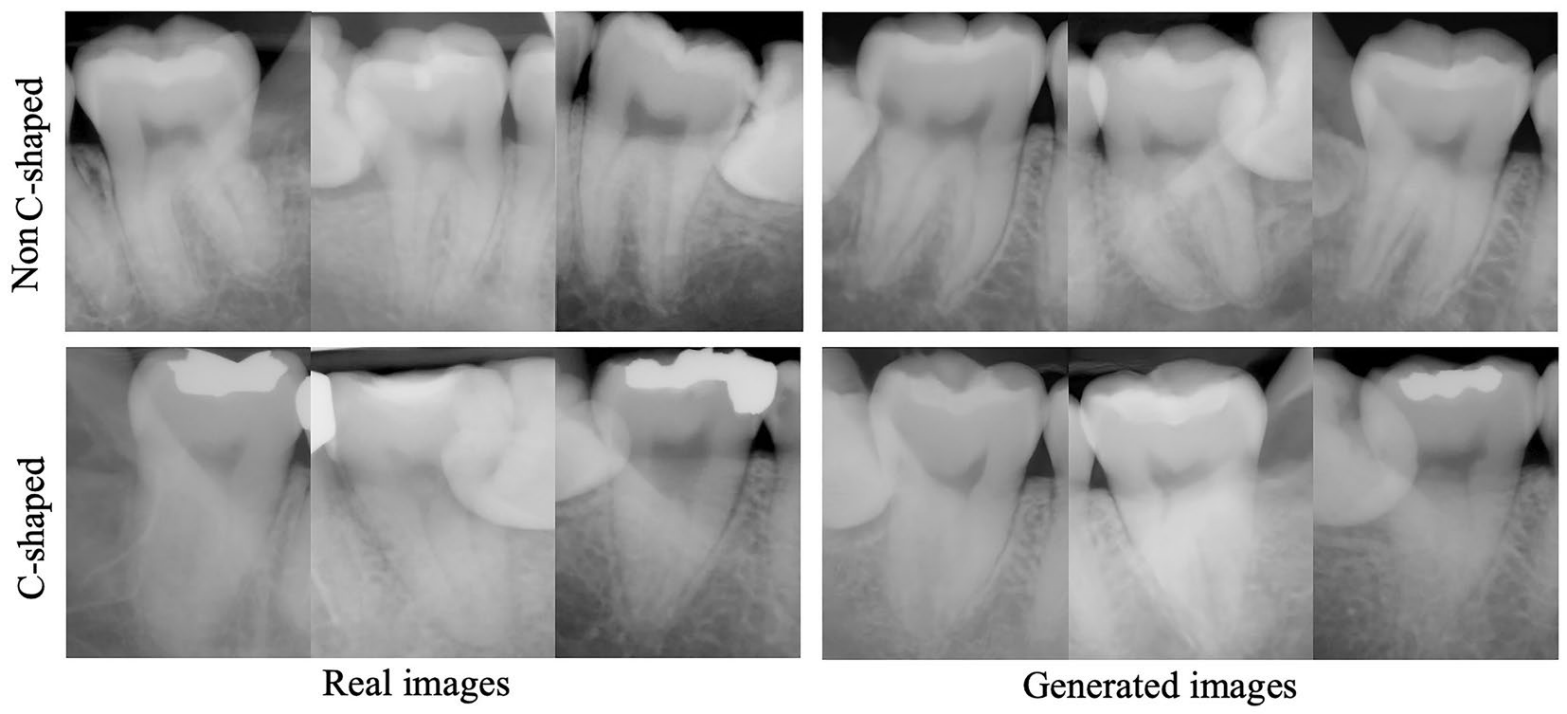
Examples of qualitative comparison between the original periapical images and GAN synthesized images having C-shaped or non C-shaped canal configuration. Some generated images show lower precision in the apical area with features such as fuzzy appearing apexes or periodontal ligaments, and less prominent root canals.

The receiver operating characteristic (ROC) curves and corresponding AUROC values for scenarios A to F are displayed in separate plots in Fig. 2. The AUROC values for scenario A to F were 0.87, 0.78, 0.92, 0.93, 0.90 and 0.94 respectively. The chi-square test yielded values of 4.114 (scenarios A vs. D) and 4.326 (scenarios E vs. F), with corresponding *p* values of 0.042 and 0.037, indicating a statistically significant difference in model performance. However, the chi-square value was 2.630 with a corresponding *p* value of 0.104 for scenario C versus D, indicating no significant difference. Examples of qualitative comparison between the original periapical images and GAN synthesized images having C-shaped or non C-shaped canal configuration are shown in Fig. 3.

## Discussion

In recent years, the use of GANs has become increasingly popular in a wide range of applications within the medical and dental fields, including image and video synthesis as well as text generation. The ability of GANs to generate realistic and diverse data samples has made GANs an important tool in machine learning and artificial intelligence research, and their potential use in dental radiography is especially compelling because of the limited availability of high-quality dental radiographs for research and training purposes. In this study, we aimed to evaluate the feasibility and effectiveness of using GANs to generate synthetic periapical radiographs and evaluate their performance in image quality and prediction outcomes.

In our study, the FID scores for the generated images from a pretrained StyleGAN2-ADA were 72.762 (± 0.723) and 61.373 (± 3.035) for radiographs with C and non-C canal configurations, respectively. These results may appear unsatisfactory when compared with other medical studies. One study^21^ reported FID scores of 5.22 (± 0.17) for a liver CT dataset on a StyleGAN2 network with transfer learning from the FFHQ dataset, and FIDs of 10.78, 3.52, 21.17, and 5.39 on the publicly available SLIVER07, ChestX-ray14, ACDC, and Medical Segmentation Decathlon (brain tumors) datasets. In another study^45^, the FID was approximately 20 for synthesized magnetic resonance and CT images. Nevertheless, the synthesized images in this study still proved useful in data augmentation and yielded good results in the visual Turing test and classification performances. The visual Turing test showed that the synthesized images had realistic and diverse characteristics (average accuracy = 0.54). Furthermore, the performance results from the EfficientNet classification indicate that the images generated from StyleGAN2-ADA could serve as a useful database for data augmentation. On the other hand, it is worth noting that while the authors were able to produce images with acceptable qualities after sufficient training; the root tips and periodontal ligaments in the apical portion shows a relatively fuzzy appearance in the generated images using StyleGAN2-ADA compared to real periapical images, and the root canals also seem less prominent. The overall results of this study show that the generated images have sufficient image quality for the diagnosis of the C-shaped canal anatomy for both human observers and the CNN; yet these call for improvement in future studies.

There may be some limitations in applying FID in medical images because the ImageNet dataset does not contain medical images. Thus, some studies argue that using FID for medical imaging is neither practical nor feasible and suggest replacing the inception network with their own encoding networks^46,47^. Nonetheless, recent studies using StyleGAN2 have reported their results using FID^21,45^, which is different from the approach of using their own encoding networks for FID evaluation in medical imaging. This is because the alternative approach lacks consistency in evaluating and comparing FID because it does not use the same encoding model as ImageNet^21,48^. For these reasons, the original FID was used as the calculating metric of this study.

Despite the promising results of this study in using GAN-generated data to improve the performance of dental radiographic image classification models, there are several limitations that should be acknowledged. First, the lack of interpretability and explainability of GAN models can be a significant obstacle to the clinical application of these models. GANs consist of a generator network that creates synthetic data and a discriminator network that distinguishes between real and fake data. The generator network uses a random noise input to produce data that resembles the real data, and the discriminator network is trained to differentiate between the real and synthetic data. Thus, learning a disentangled representation is still a challenge in the field of the interpretability of GANs, and identifying errors or biases in the generated data is challenging. Studies seeking methods to modify traditional GANs to ensure explainability and interpretability by manipulating feature or saliency maps of the corresponding filters are showing promising results^49,50^; therefore, implementing interpretable GANs must be considered in future studies for stable image generation and increased applicability in the clinical field.

Second, the evaluation of generative models lacks an objective loss function, making it necessary to assess the quality of the generated synthetic images. Although manually inspecting and judging the generated examples at different iteration steps is a basic and useful approach to evaluate a GAN, this method has limitations because it is basically subjective and may include the biases of the reviewer. Additionally, it requires domain knowledge to distinguish between realistic and unrealistic images, making it essential to involve experts in the field. Furthermore, the number of images that can be reviewed is limited by the capacity to undertake manual inspection, and no clear best practice has emerged for qualitatively assessing the generated images, as it is likely to vary depending on the specific case. In this study, it was particularly important to rely on the assessments of dental specialists when evaluating the synthetic periapical radiographs. More methods that can objectively evaluate GANs are needed in future GAN implementations for image data augmentation.

Third, the high cost of model training and the need for a larger and diverse dataset are also significant challenges to consider. This study used a dataset of 1400 images—a relatively small number when compared with other studies—and the model was trained using a single GPU. Although the authors of StyleGAN2-ADA suggest that the model produces promising results from a small dataset of over 1000 images, the amount of data needed is still unclear; therefore, a larger and more diverse dataset from different institutions is strongly recommended for better generation outcomes. Additionally, further studies using external datasets are needed to evaluate the generalizability of the proposed method. Within the GAN training dataset of this study, the truncation was manually set (Ψ = 1, 0.7, 0.5, − 0.5, − 0.7, − 1) to generate moderately diverse yet stable results and avoid mode collapse. Generated images also went through manual screening by looking at the thumbnails, which implies that generated images can seem unrealistic in some cases despite their visual quality and must be inspected by experts. An alternative that can generate realistic and diverse data in a more stable and automatic manner and within limited computational resources should be implemented in future studies. In this study, the image size was limited to 512 × 512 pixels, and the region of interest was manually cut out. Future work should focus on the full periapical image synthesis.

Fourth, the effect of adding GAN data must be further recognized. In general, incorporating GAN-generated images into the real dataset led to an improvement in classification performance, indicating that GAN-generated data can serve as an effective method for data augmentation. However, when examining the ROC, training the CNN solely with GAN-synthesized data (scenario B) displayed a lower true positive rate compared with the CNN trained solely with real data (scenario A) or augmented data (scenario C, D). Nonetheless, the false positive rate was found to be similar between the two approaches. Notably, the true positive rate increased as more data was added, regardless of whether it was real data or GAN-generated data. The low true positive rate observed when training the CNN with GAN-synthesized data suggests that the generated images might not accurately capture the specific features necessary for correctly identifying C-shaped canals. The GAN-generated data may lack certain details or exhibit variations that make it more challenging for the CNN to accurately classify C-shaped canals. However, the similar false positive rate between the CNN trained with GAN-synthesized data and the CNN trained with real data suggests that the GAN data does not significantly contribute to an increased number of false positives. This might indicate that the GAN-synthesized images do not introduce additional incorrect classifications beyond those already produced by the CNN trained with real data. Overall, these results suggest that the GAN-synthesized data might not be fully representative of the specific characteristics required for accurate classification of C-shaped canals, but up to this extent can serve as an effective data augmentation method in both situations with or without data imbalance. This conclusion is supported by the observation that the chi-square scores were 4.114 (scenarios A vs D) and 4.326 (scenarios E vs F), with corresponding p-values of 0.042 and 0.037. Additionally, the ROC and AUC values are comparable when comparing scenarios where data augmentation was performed using traditional methods (scenario C) versus adding GAN-synthesized data (scenario D). Therefore, within this context, GAN-generated data offers a viable approach for enhancing the training dataset.

Finally, while GANs have shown great potential for image generation tasks in dentistry, their use in combination with other deep learning tasks in the field remains largely unexplored. In medicine, the use of GANs has been thoroughly investigated, not only in data augmentation^11^ but also in image segmentation^12^, denoising and super-resolution, domain transfer, and post intervention prediction^51^. Therefore, the development momentum of GAN-based dental deep learning studies must be updated and reinforced, and future studies should investigate the potential benefits of using GANs in combination with other deep learning approaches by conducting cooperative studies with multiple facilities.

## Conclusion

In conclusion, the results of this study suggest that the periapical images generated using StyleGAN2-ADA may not fully capture the precise characteristics required for accurate classification of C-shaped canals; however, these generated images exhibit satisfactory visual quality and demonstrate potential as an effective data augmentation method. The use of GAN-generated images can help overcome the challenges associated with insufficient training data and limited computational resources in diagnosing dental anomalies or diseases. Future studies are anticipated to enhance the quality of GAN-generated periapical images by incorporating larger and more diverse datasets that encompass a broader range of anatomical and disease features.

### Supplementary Information

Supplementary Information.

## Data Availability

The datasets are not publicly available. Ethics approval for using the de-identified images of this study will be provided upon request to the corresponding author.

## Abbreviations

GANs: Generative adversarial networks
CNNs: Convolutional neural networks
ADA: Adaptive discriminator augmentation
