# Supplementary material for: Evaluating the performance of generative adversarial network-synthesized periapical images in classifying C-shaped root canals
Source: Sci Rep. 2023 Oct 21;13:18038. doi: 10.1038/s41598-023-45290-1 (PMC10590373; doi:10.1038/s41598-023-45290-1)
Supplement: Supplementary file 1 — Supplementary Information. [file 41598_2023_45290_MOESM1_ESM.docx]

Supplementary Table 1. T-test results between scenarios A to F. The significance level (alpha) was set to 0.05.

|  | Accuracy | Precision | Recall | Specificity | FPR | FNR |
| --- | --- | --- | --- | --- | --- | --- |
| A vs B | 0.2632 | 0.3461 | 0.9023 | 0.4215 | 0.4215 | 0.9023 |
| A vs C | 0.4909 | 0.7670 | 0.9062 | 0.5746 | 0.5746 | 0.9062 |
| A vs D | 0.1239 | 0.6918 | 0.1609 | 0.6370 | 0.6370 | 0.1609 |
| A vs E | 0.9352 | 0.3598 | 0.1079 | 0.4188 | 0.4188 | 0.1079 |
| A vs F | 0.2577 | 0.7211 | 0.2756 | 0.9385 | 0.9385 | 0.2756 |
| B vs C | 0.0998 | 0.0901 | 1.0000 | 0.0753 | 0.0753 | 1.0000 |
| B vs D | 0.0310* | 0.0722 | 0.1161 | 0.0735 | 0.0735 | 0.1161 |
| B vs E | 0.2972 | 0.7082 | 0.0815 | 0.8297 | 0.8297 | 0.0815 |
| B vs F | 0.0817 | 0.2207 | 0.1543 | 0.2048 | 0.2048 | 0.1543 |
| C vs D | 0.0533 | 0.6749 | 0.0341* | 0.7040 | 0.7040 | 0.0341* |
| C vs E | 0.5078 | 0.1597 | 0.0186* | 0.1328 | 0.1328 | 0.0186* |
| C vs F | 0.7040 | 0.1456 | 0.1027 | 0.0777 | 0.0777 | 0.1027 |
| D vs E | 0.2441 | 0.2150 | 0.0705 | 0.2103 | 0.2103 | 0.0705 |
| D vs F | 0.2056 | 0.1959 | 1.0000 | 0.2455 | 0.2455 | 1.0000 |
| E vs F | 0.3950 | 0.2905 | 0.3739 | 0.2312 | 0.2312 | 0.3739 |
